# Small mammal diversity of Mt. Kenya based on carnivore fecal and surface bone remains

**DOI:** 10.24272/j.issn.2095-8137.2018.055

**Published:** 2018-10-16

**Authors:** Ogeto Mwebi, Esther Nguta, Veronica Onduso, Ben Nyakundi, Xue-Long Jiang, Esther N. Kioko

**Affiliations:** 1 Osteology Section, Department of Zoology, National Museums of Kenya, Nairobi 40658-00100, Kenya; 2 Kunming Institute of Zoology, Chinese Academy of Sciences, Kunming Yunnan 650223, China; 3 Sino-African Joint Research Center, Chinese Academy of Sciences, Nairobi 62000-00200, Kenya

**Keywords:** Ecological dynamics, Faunal diversity, Scats, Pellets, Mt. Kenya

## Abstract

Ecological dynamics and faunal diversity documentation is normally conducted by direct observation and trapping of live animals. However, surveys of carnivore scat prey and surface bone remains, which are relatively inexpensive, can provide complementary data that expand carnivore diet breadth and may improve accuracy regarding inferences of the ecological dynamics of a given ecosystem. We used this inexpensive method to document species diversity variation with elevation on the leeward (Sirimon) and windward (Chogoria) areas of Mt. Kenya. Bone and fecal specimens were opportunistically collected by walking 2 km in opposite directions from transect points selected at 200-m intervals along the elevational gradient of the study areas. We collected a total of 220 carnivore fecal and owl pellet specimens from both study sites, which were mainly deposited by the spotted hyena (*Crocuta crocuta*), leopard (*Panthera pardus*), serval (*Leptailurus serval*), genet (*Genetta* sp.), and Mackinder’s Cape owl (*Bubo capensis mackinderi*). Serval scats were the most common, followed by those of the spotted hyena. Scats and bones were found at the lowest density at the lowest elevations, peaked at mid-higher elevations, and then declined at the highest elevations. Based on skeletal analysis only, there were more species in Sirimon (19) than in Chogoria (12). Small fauna (rodents to duiker size bovids) formed the bulk of the identified remains, representing 87.9% of the Sirimon fauna and 90.9% of the Chogoria fauna. The genus *Otomys* was the dominant prey of the owl and serval in both sites. Three giraffe teeth were found at 3 500 m a.s.l. in Chogoria on the edge of Lake Ellis, suggesting that it is an occasional visitor to such high elevations. This study underscores the value of fecal and bone surveys in understanding the diet and diversity of mammals in ecological ecosystems, but such surveys should be complemented with analysis of hairs found in scats to obtain a more complete list of carnivore prey at Mt. Kenya.

## INTRODUCTION

Caves and rock shelters, which are suitable for carnivore lairs and bird of prey roosting sites (e.g., for owls), are locations where bones and indigestible material like hair accumulate and can thus serve as sources for faunal diversity documentation (Behrensmeyer & Miller, 2012; Shaw, 1979; Terry, 2010). Not only do bone assemblages reveal the identity and behavior of the accumulators by the signatures they leave on them, but they also highlight food resources, population dynamics and environmental conditions of the area (Behrensmeyer & Miller, 2012; Kerbis-Peterhans, 1990; Klein & Cruz-Uribe, 1984). Thus, considerable information can be obtained from examination of the indigestible/inedible remains of carcasses that predators have discarded. Bone representation may indicate mortality due to natural causes and or hunting pressure and the level of their destruction by predators may indicate food resource scarcity/availability (Faith et al., 2007). For example, studies on the Amboseli ecosystem have shown strong correlation between bone assemblages and the living vertebrate community (Behrensmeyer, 1978; Behrensmeyer & Boaz, 1980; Western & Behrensmeyer, 2009). Thus, this inexpensive method can complement standard biodiversity surveys by contributing additional data to aid in our understanding of the ecological dynamics of a given ecosystem.

Documentation of faunal diversity and ecological dynamics is normally undertaken by direct observation and trapping of live animals. However, sightings of small and some migratory vertebrates can be difficult and unpredictable. This is particularly so where thick vegetation and lack of appropriate equipment hampers visibility and when the timing of the field work is wrong and limited. Thus, direct observation alone may not provide complete information on the fauna of a given area. Carnivore fecal deposits in caves or rock shelters (that serve as lairs or roosting sites) accumulate bones and other indigestible prey remains. Remnants of rare and cryptic animals are likely to be deposited by their predators in such areas. If bone or hair remains of such animals are found, they can indicate their occurrence in the area. For example, Sillero-Zubiri et al. (1995) reported on the Ethiopian wolf (*Canis simensis*) diet in the Bale Mountains, with *Otomys typus* found to be well represented in wolf scats in areas where the rodent had never been trapped during standard rodent surveys. Similarly, in another study in Africa, species that were unknown from live census data were found in bone assemblages, including domestic animals that were illegally brought into national parks to graze (Behrensmeyer & Miller, 2012). Therefore, analysis of faunal remains can fill in gaps in our knowledge of faunal diversity in an ecosystem and complement standard faunal surveys, which can be time consuming and expensive (Behrensmeyer & Miller, 2012). In addition, standard surveys only focus on animals that are currently present in an area and may not detect recent local extinctions. Given the right conditions, prey remains (hairs and bones) may be preserved for decades or centuries and are likely to record fauna that may have become locally extinct. Detection of local extinctions is important in reconstructing any environmental changes taking place.

While analysis of carnivore remains can capture data missed by standard surveys, it does present potential biases. For example, seasonal prey availability can bias results when prey available in a given season is the only one eaten and discarded. Analysis of such remains will exclude species from other seasons. Similarly, predator prey preferences can result in the preferred prey being detected in the discarded remains, whereas those that are ignored are not found. Furthermore, the size of some prey, digestive system strength of the predator, and other post-mortem processes may result in the complete digestion of prey, which will therefore not be detected in the discarded remains. Thus, taphonomic histories of remains and prey selection of the predators must be considered for accurate interpretation of how the remains represent living communities of fauna in a given area (Terry, 2010). However, several studies have reported close correspondence between skeletal remains analysis and live census species richness and relative abundance (Behrensmeyer & Miller, 2012; Miller et al., 2014; Terry, 2010; Western & Behrensmeyer, 2009). To our knowledge, no survey of skeletal remains has been conducted on Mt. Kenya with the aim to document variation in its altitudinal faunal diversity. Previous studies have only focused on the reconstruction of bird of prey diets through analysis of the skeletal remains in their pellets (Rödel et al., 2002). Thus, we conducted a 40-day survey of animal remains on Mt. Kenya (Chogoria and Sirimon areas) from 3 September to 13 October 2015 in the dry season and collected carnivore prey bones and scats for identification and analysis. We aimed to use skeletal and other animal hard tissue remains in carnivore scats to document species diversity variation with elevation. Given that no such survey using this method has been conducted in this study area, the data collected will serve as a baseline against which future studies can be developed.

## MATERIALS AND METHODS

### Study area

This study was conducted along the windward (Chogoria, eastern slopes) and leeward (Sirimon, western slopes) zones of Mt. Kenya during the dry season. The mountain lies between S0°10′, E37°20′, and rises from 1 600–5 200 m a.s.l. (Bennun & Njoroge, 1999). It is located in central Kenya and spans five counties (Meru, Embu, Kirinyaga, Laikipia, and Nyeri). The lower slopes are covered by mixed indigenous forest (from 2 000 m to 2 400 m and 2 400 m to 2 600 m a.s.l. on the eastern and western slopes, respectively), except at 1 800 m a.s.l. on the eastern slopes where there is a mixed plantation of *Eucalyptus* and *Grevellia* with indigenous trees. The indigenous forests then give way to bamboo forests from 2 600 m to 2 800 m a.s.l. on the eastern slopes and mixed bamboo forests at 2 800 m a.s.l. on the western slopes. *Juniperus-Hagenia* habitat, with the canopy dominated by *Hagenia abyssinica*, is found at 3 000 m a.s.l. on the eastern slopes and from 3 000 m to 3 200 m a.s.l. on the western slopes. Montane grassland and heather or alpine zones dominated by *Erica* bushes range from 3 200 m to 3 600 m a.s.l. on the eastern slopes and from 3 400 m to 4 200 m a.s.l. on the western slopes (Kioko et al., 2016; Musila et al., 2019). These vegetation zones are more clearly defined in the Chogoria site than the Sirimon site, which has a mixture of *Erica* and bamboo along its elevational gradient from the lower forest zone (Malonza, 2015). The lower slopes of the mountain are cultivated up to 1 800 m a.s.l. in the south, 2 400 m a.s.l. in (some) eastern and western areas, and 2 900 m a.s.l. on the northern slopes (Bussmann, 2006).

### Scat and bone surveys

**Figure 1:**
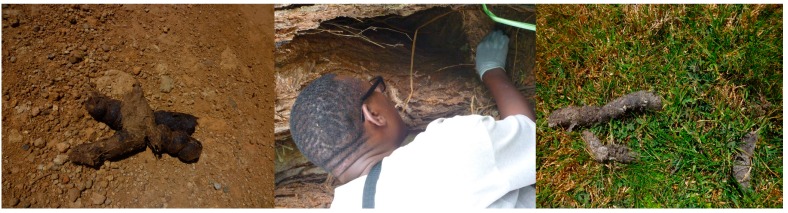
Leopard scat on road (left) and cave (middle) and serval scat on short grass (right)

Sampling points were marked at 200-m elevational intervals along a transect (roads or already established mountain climbing trails) in both study sites beginning from the lowest elevation of the mountain where a forest begins to the alpine zone (1 800 m to 3 500 m a.s.l. for Chogoria and 2 400 m to 4 200 m a.s.l. for Sirimon) using a Geographical Positioning System (GPS) unit. Bone and fecal specimens were opportunistically collected by two survey groups walking 2 km in the opposite direction (east-west) from each sampling point on the road/trail. Thorough random searches were conducted along animal trails, with researchers inspecting under trees suitable as roosts for birds of prey and searching caves whenever they were found. Local people were interviewed to gather information on any known owl roost sites and carnivore dens. Walks along each transect (cutting through all sampling points) were also done and any deposited feces were collected. Once found, the feces or bones (whether old or fresh) were photographed *in-situ* and then collected, separately bagged, and labelled with their location point and date of collection (Figure 1). Whenever possible a preliminary identification of the species that deposited the scat was recorded. Carnivore feces, as opposed to those of herbivores, are normally packed with indigestible prey bones, hairs, hooves, claws, and feathers, and their identification was based on their presence within the feces. Identification of the depositing carnivore was based on the known size and shape of each carnivore’s feces and confirmed by the known distribution of the carnivore in the study site through interviews with local people and from the literature (Kingdon, 1977). The collected specimens were taken to the National Museums of Kenya Osteology Section Laboratory for identification and storage.

In the laboratory, the scats were identified following Chame (2003) and Stuart & Stuart (2000). The skeletal elements of each scat/pellet were separated and sorted by hand. Before separation and sorting they were soaked in alcohol overnight for sterilization and then soaked in warm water (after draining the alcohol) for several hours until they were soft enough to be separated without breaking the bones. The soft scat was thoroughly rinsed in a fine sieve using flowing cold tap water. The wet rinsed scat was then transferred from the sieve onto a plastic tray and the skeletal remains were carefully picked by hand and forceps and placed onto a tray for identification. The hair and debris from each scat/pellet were dried and separately bagged and stored for future identification and analysis.

### Data analysis and scat identification

The dry bone remains were identified using comparative material housed in the Osteology Section of the Zoology Department at the National Museums of Kenya. For small mammals whose comparative material was not available, only crania and mandibles were identified using dentition, following the identification keys described in Happold (2013). We determined the minimum number of individuals (MNI) per scat following Klein & Cruz-Uribe (1984) and Lyman (2008) and species/scat abundance was expressed as a percentage of occurrences (Geffen et al., 1992; Rödel et al., 2002). Given the fragmentary nature of the skeletal remains, it was not possible to identify all specimens to species level.

## RESULTS

A total of 220 fecal and owl pellet specimens packed with prey hairs and bones from both study sites (116 in Chogoria and 104 in Sirimon) were collected. However, no owl pellets were found in Chogoria. We identified 93.1% of the skeletal remains from Sirimon and 69.7% of those from Chogoria to at least genus level. The MNI counts for Sirimon and Chogoria were 124, representing 19 species, and 88, representing 13 species, respectively. Of the 19 species from Sirimon, 12 were from scat/pellet skeletal remains and seven were from non-scat skeletal remains, whereas eight of the 13 species from Chogoria were identified in scats and five from non-scat skeletal remains (Table 1). All non-scat remains had predator teeth marks, which indicated that they were either scavenged or killed by a predator. However, two of the remains (a Sykes monkey and a Jackson’s francolin) in Sirimon were non-scavenged road kills.

**Table 1:** List of species, minimum number of individuals (MNI), and proportion in the study areas

			Sirimon		Chogoria	
Family	Species	Source	MNI count (*n*)	Proportion (%)	MNI count (*n*)	Proportion (%)
Muridae	*Acomys* sp.	Scat	N/A	N/A	13	14.8
Muridae	*Lemniscomys* sp.	Scat/Owl pellet	5	4.0	1	1.1
Muridae	*Thamnomys* sp.	Scat	1	0.8	N/A	N/A
Muridae	*Rhabdomys* sp.	Scat/Owl pellet	12	9.6	N/A	N/A
Muridae	*Mus* sp.	Scat	N/A	N/A	1	1.1
Cricetidae	*Otomys* sp.	Scat/Owl pellet	61	49.2	43	48.9
Rhizomidae	*Tachyoryctes splendens*	Scat	1	0.8	19	21.6
Leporidae	*Lepus* sp.	Scat	2	1.6	N/A	N/A
Soricidae	*Crocidura* sp.	Scat	4	3.2	N/A	N/A
Hyracoidea	*Procavia* sp./*Dendrohyrax arboreus*	Scat	5	4.0	1	1.1
Phasianidae	*Francolinus* sp.	Scat/Owl pellet	3	2.4	N/A	N/A
Phasianidae	*Francolinus jacksonii*	Road kill	1	0.8	N/A	N/A
Turdidae	*Turdus* sp.	Owl pellet	4	3.2	N/A	N/A
Ardeidae	*Ardea melanocephala*	Bone scatter	N/A	N/A	1	1.1
Cercopithecidae	*Cercopithecus mitis*	Road kill	2	1.6	N/A	N/A
Bovidae	*Cephalophus* sp.	Scat	7	5.6	1	1.1
Bovidae	*Sylvicapra grimmia*	Scat	1	0.8	N/A	N/A
Bovidae	*Kobus ellipsiprymnus*	Bone scatter	3	2.4	1	1.1
Bovidae	*Syncerus caffer*	Bone scatter	5	4.0	2	2.3
Bovidae	*Taurotragus oryx*	Bone scatter	1	0.8	N/A	N/A
Bovidae	*Tragelaphus scriptus*	Bone scatter	3	2.4	3	3.4
Equidae	*Equus quagga*	Bone scatter	3	2.4	N/A	N/A
Giraffidae	*Giraffa camelopardalis*	Bone scatter	N/A	N/A	1	1.1
Hyaenidae	*Crocuta crocuta*	Bone scatter	N/A	N/A	1	1.1
Total MNI count			124		88	

N/A: Not available.

**Figure 2:**
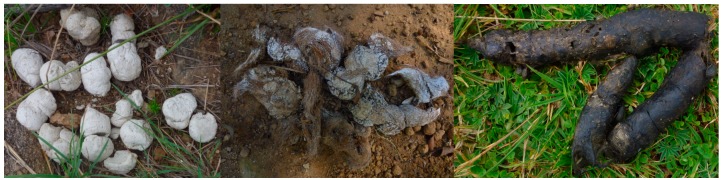
Hyena (left), leopard (middle), and serval (right) scats, respectively

The scats were deposited by the spotted hyena (*Crocuta crocuta*), leopard (*Panthera pardus*)*,* serval (*Leptailurus serval*) (Figure 2), and genet (*Genetta* sp.), whereas the pellets were deposited by the Mackinder’s Cape owl (*Bubo capensis mackinderi*). The serval scats formed most of the collections, followed by owl pellets. The leopard and hyena scat proportions were lower (7.7% and 11.2% of total Chogoria scats, respectively) in Chogoria compared to Sirimon (11.9% and 20.9% of total Sirimon scats, excluding owl pellets, respectively). In addition, 88.9% of leopard and 62.5% of genet scats were found along the road in the forested lower elevations (2 200 m to 3 000 m a.s.l.), whereas those of the serval (92.9%) and hyena (84.6%) were found at higher elevations (3 000 m to 3 500 m a.s.l.) in the open grassland (alpine zone/moorland) in Chogoria. The reverse was the case for the leopard in Sirimon, where all (100%) its scats were found at higher elevations (3 600 m to 4 200 m a.s.l.).

**Figure 3:**
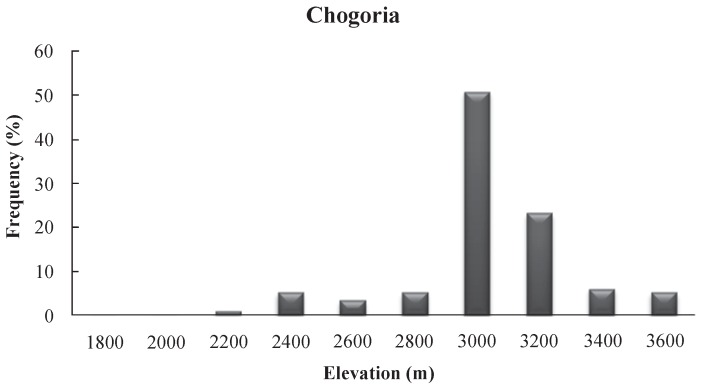
Carnivore scat frequency of occurrence with elevation in Chogoria

**Figure 4:**
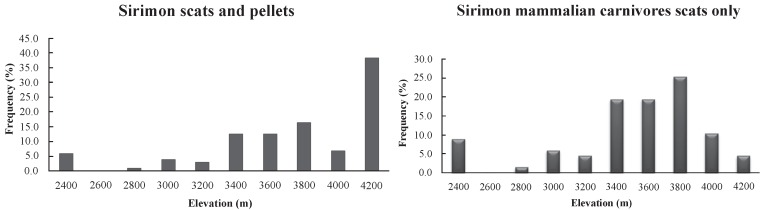
Carnivore scat frequency of occurrence, including owl pellets (left) and excluding owl pellets (right), with elevation in Sirimon

The mammalian carnivore scat deposit frequencies increased with elevation to the alpine zone/moorland (3 000 m to 3 200 m a.s.l. in Chogoria and 3 400 m to 3 800 m a.s.l. in Sirimon), where they were highest, and then decreased at higher elevations (Figure 3 and Figure 4). The high numbers of pellets at 4 200 m a.s.l. in Sirimon were the result of a single roost site accumulated over a long period of time.

**Figure 5:**
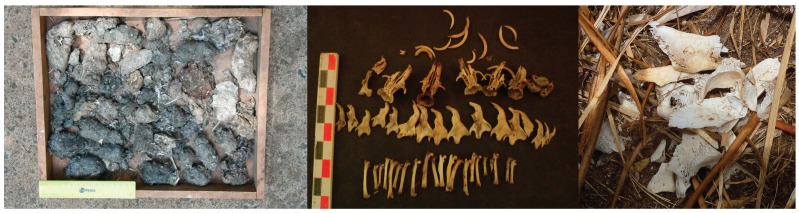
Owl pellets (left), rodent remains from owl pellets (middle), and bone remains from hyena regurgitation (right)

The identification level of prey species in the remains varied with the species that deposited them and whether they were scat or non-scat remains. Non-scat remains mainly consisted of large, easily identified mammals and birds, whereas identification of scat remains depended on the carnivore that deposited them. Leopard and hyena scats consisted of mainly hairs and highly fragmented bones, which were not possible to identify. In contrast, serval scats and owl pellets contained small mammal and bird remains that were not highly fragmented (some elements were complete), thus allowing easier identification (Figure 5). Therefore, over 80% of the recognized species were small mammals and birds (reptiles and fish were not found in any remains from either site) identified in serval scats and owl pellets from both study sites.

Small fauna (rodents to duiker size bovids) formed the bulk of the identified remains, representing 87.9% of the Sirimon fauna and 90.9% of the Chogoria fauna. Of the mammals, the rodents dominated, accounting for 66.1% of the identified Sirimon remains and 87.5% of the identified Chogoria remains. *Otomys* sp. was the most common rodent found in the scats/pellets, occurring in the moorland/alpine zones dominated by tussock grasslands in both areas, and represented 48.9% of the identified individuals in Sirimon scats from 3 400 m to 4 200 m a.s.l. and 49.2% of the identified individuals in Chogoria scats from 2 800 m to 3 500 m a.s.l. at Lake Ellis. *Acomys* sp., *Otomys* sp., and *Tachyoryctes splendens* were the most frequent rodents found in Chogoria scats, accounting for 85.3% of identified remains, whereas *Otomys* sp. was the single most common rodent in owl pellets and serval scats in Sirimon, representing 48.9% of the identified individuals, followed by *Rhabdomys* sp. at 9.6%, which was identified in scats at 2 400 m, 3 000 m, and 3 400 m a.s.l.. Bird remains were uncommon in both study areas but more individuals were identified in Sirimon remains (eight individuals), representing 6.5% of the total identified individuals in the area, compared with Chogoria remains (one individual), representing 1.1% of the total identified individuals in the area. Large mammals ranging from bovid II size class/impala size and above (including spotted hyena) were also uncommon in the remains, with five species identified in Sirimon, representing 12.1% of the total individuals, and five species identified in Chogoria, representing 9.1% of the total identified individuals (Table 1).

## DISCUSSION

In our study, prey remains and carnivore scats varied between the three (forest, moorland, and alpine) mountain zones. Encounters were low at the lowest forested elevations, with no scat or prey remains collected in the mixed forest at the 1 800 m and 2 000 m a.s.l. sampling points along the Chogoria transect or at the 2 600 m a.s.l. sampling point along the Sirimon transect. This is consistent with the assumption that prey density is low and scattered in dense forest, which makes prey encounters by predators unpredictable, and thus predators tend to avoid forests compared to grasslands (Farrell et al., 2000). However, scat abundance tended to increase with altitude in the Chogoria forest zone (1 800 m to 2 800 m a.s.l.), though the reverse was true for the Sirimon forest zone (2 400 m to 2 800 m a.s.l.). Only six scats, from which five individuals of three small mammal species were identified, were located at the 2 400 m sampling point in Sirimon, whereas 17 scats, from which 12 individuals of five species were identified, were collected from sampling points from 2 200 m to 2 800 m a.s.l. in Chogoria. The Sirimon forest zone trend is consistent with the common tropical montane forest pattern where vertebrate diversity tends to decrease with altitudinal increase (Clausnitzer & Kityo, 2001; Tuyisingize et al., 2013). However, this pattern is not always the same due to the influence of several biotic and abiotic factors, including level of forest disturbance (Bertuzzo et al., 2016; Clausnitzer & Kityo, 2001; McCain & Grytnes, 2010; Tuyisingize et al., 2013). The species diversity index was higher in the Chogoria (1.52) forest zone than that in Sirimon (0.95). Given that there was no difference in the carnivore species (spotted hyena, serval, and genet were found in both areas) scats collected along both transects, the observed differences in species diversity were likely the result of forest disturbance. Forest disturbance at the higher altitudes in Chogoria is less than that in Sirimon, leading to the greater diversity of small mammal species observed at Chogoria than that of Sirimon. Bussmann (2006) observed forest disturbance from agricultural activities up to 2 900 m a.s.l. along the Sirimon transect. Malonza (2015) studied the herpetofauna of the two transects and attributed the high species diversity of Chogoria to its windward location, thus receiving more rainfall and experiencing higher productivity. A similar species diversity trend persisted even up to the open *Hagenia* forest at 3 000 m a.s.l. of the study sites with the Chogoria site having a higher species diversity than Sirimon.

Species diversity remained low (0.69 Shannon H index) even at the *Hagenia* open forest zone at 3 000 m a.s.l. (above the bamboo forest line) of the Sirimon transect, whereas that along the Chogoria transect was higher (1.11). Four scats attributed to spotted hyena, serval, and genet containing two prey species (*Procavia* sp. and *Rhabdomys* sp.) were collected in Sirimon, whereas 59 scats attributed to spotted hyena, leopard, serval, and one unknown carnivore were collected in Chogoria. The low number of species identified in this Sirimon zone is likely a function of our methodology because concurrent trapping conducted by the mammalogy team during this study (Musila et al., 2019) captured 16 species of small mammals. However, the small number of carnivore scats collected in this zone suggests low carnivore activity in the area compared to the same zone in Chogoria. As discussed below, little research has documented the mammalian diversity of these zones at both study sites. To date, more focus has been directed on the alpine/*Ericaceous* zone of the Sirimon site (e.g., Coe, 1967; Coe & Foster, 1972; Moreau, 1944; Sessions, 1972; Young & Evans, 1993). This open woodland had the highest number of scats and species (eight) from the entire Chogoria transect but its species diversity index was lower (1.11) than that of the alpine/*Ericaceous* zone (1.57) because about 70% of the total individuals identified here were one species (*Otomys* sp.).

In the ericaceous zone, mammalian carnivore scat abundance tended to increase with altitude, but dropped at the highest altitude (4 000 m a.s.l. for Sirimon and 3 500 m a.s.l. for Chogoria) in both study sites. This zone contained a total of 56 (46 individuals of eight species) and 40 (26 individuals of seven species) scats collected from Sirimon and Chogoria, respectively. Despite having almost the same number of species, the diversity index of the Chogoria zone was higher (1.57 vs. 1.22) because three of its abundant species (*Acomys* sp., *Tachyoryctes splendens*, and *Otomys* sp.) were represented by almost the same number of individuals. On the other hand, in the Sirimon zone, *Otomys* sp. was the single most abundant species, representing 65% of the total individuals. *Otomys* sp. is a well-documented major prey of carnivores and the most abundant species of the alpine zone (Bertuzzo et al., 2016; Clausnitzer & Kityo, 2001; Coe, 1967; Coe & Foster, 1972; Moreau, 1944; Sessions, 1972; Tuyisingize et al., 2013; Young & Evans, 1993). All scats of this zone were attributed to the same mammal carnivores (spotted hyena, leopard, serval, and genet) in both study sites.

Leopard scats were absent at the highest (4 200 m a.s.l.) elevation sampled in Sirimon, even though this species has been reported to occur up to 4 800 m a.s.l. on this side of the mountain preying on hyrax, colobus, and *Otomys* sp. (Rödel et al., 2004; Young & Evans, 1993). We also did not find any scats attributable to the cheetah (*Acinonyx jubatus*) or African wild dog (*Lycaon pictus*), despite their reported occurrence in the alpine zone (Young & Evans, 1993), because the harsh alpine temperature conditions at the highest elevations likely influenced the abundance of both predators and prey. However, the large number of owl pellets containing *Otomys* sp. collected at 4 200 m a.s.l. in the Sirimon study site confirmed that certain prey and their predators are adapted to the harsh cold alpine conditions. *Otomys* sp. is particularly common in the alpine zones of the mountains in Africa and is commonly found in scats/owl pellets of the zone’s predators (e.g., Bertuzzo et al., 2016; Clausnitzer & Kityo, 2001; Coe, 1967; Coe & Foster, 1972; Moreau, 1944; Rödel et al., 2002, 2004; Sessions, 1972; Tuyisingize et al., 2013; Young & Evans, 1993). In general, large herbivore and carnivore remains were rare in both study areas. This reflects the low populations of these vertebrates in the alpine ecosystem.

An interesting find in relation to herbivores was the giraffe at 3 500 m a.s.l. in Chogoria. Even though represented by three teeth, this species is not usually known to occur at such high elevations. It is an occasional visitor to Lake Ellis (where the teeth were found) during drought. Given that there was considerable zebra dung on the lake edge, it is likely that other large herbivores visit the lake. Moreau (1944) noted that besides the hyrax and duiker, the eland is a common large moorland herbivore on the Sirimon side of Mt. Kenya and forms a major prey species. This suggests that the presence of giraffes at Lake Ellis is unsurprising. The Sirimon study area had more large herbivores, resulting in its slightly higher (1.63 vs. 1.54) overall species diversity than that of Chogoria, suggesting there is a likelihood of lower prey density in Chogoria than in Sirimon. However, these differences could be because of lower predator densities in Chogoria, resulting in less predation of the available prey.

A predator tends to be selective when its preferred prey is abundant and becomes a generalist as its preferred prey becomes scarce (Carvalho & Gomes, 2001; Cooper, 1990; Cooper et al., 1999; Farrell et al., 2000). Changes in prey abundance may be seasonal (e.g., Cooper, 1990, Darimont et al., 2008) or due to elevational prey species density variations (Coe, 1967). In this study, large herbivore prey remains were few and almost absent at the higher elevations. Thus, predators at these higher elevations subsisted on the available small herbivores (e.g., hyrax, rodents, and duikers). For example, we found *T. splendens* remains in some hyena scats, consistent with Sillero-Zubiri et al. (1995) who asserted that large mammal density in the Afro-alpine grasslands is low and that carnivores in these areas tend to specialize in hunting small mammals. In this study, small carnivore scats and owl pellets were dominant, indicating that they are the dominant predators in the alpine zone. The serval scats were especially abundant in the Chogoria grasslands, consistent with Grimshaw et al. (1995) who stated that servals prefer the moorlands of the alpine ecosystem where their scats are abundant. Coe & Foster (1972) did not mention the presence of servals on the Timau (Sirimon) side of Mt. Kenya but instead found *Felis silvestris lybica* and *Felis* (*Panthera*) *pardus* to be common moorland predators. Conversely, Moreau (1944) reported servals to be occasional visitors in the area. These discrepancies may be due to its nocturnal lifestyle, given that it was not recorded by Young & Evans (1993). We did not find any scats attributable to *Felis lybica.*

Serval scats and owl pellets dominated in the Sirimon remains collected from the alpine zone. Aside from Mackinder’s Cape owl, other birds of prey found in this alpine zone include Verreaux’s eagle owl (*Bubo lacteus*) and Lammergeyer (*Gypaetus barbatus meridionalis*), but they are not common (Coe, 1967; Moreau, 1944). Although the augur buzzard is a common bird of prey in the moorlands (Moreau, 1944; Young & Evans, 1993), we found no pellets that could be attributed to this species. The alpine zone that extends from 3 500 m to 4 570 m a.s.l. on the Sirimon study site has had more biodiversity and diet of the predators research undertaken than on the Chogoria side (e.g., Coe, 1967; Coe & Foster, 1972; Moreau, 1944; Sessions, 1972; Young & Evans, 1993). Small mammals and rodents sustain these predators, with *Otomys* sp. known to form a major part of their diet (Coe, 1967).

In the current study, *Otomys* sp. was the major food item of the Mackinder’s Cape owl and serval in Sirimon, representing 48.9% of the identified individuals, whereas in Chogoria the serval represented 49.2% of the total identified individuals. *Acomys* sp., *Otomys* sp., and *Tachyoryctes splendens* were the most common food items of the serval in Chogoria, accounting for 85.3% of the identified remains; whereas, in Sirimon, *Otomys* sp. was the single most important food item of the owl and serval. In a study of pellets in the alpine zone at Kazita Valley of Mt. Kenya west of Hook Tarn, 92% and 92.5% of augur buzzard and Mackinder’s owl pellets, respectively, contained *Otomys* sp. (Coe & Foster, 1972). Similarly, Rödel et al. (2002) found that *Otomys* sp. accounted for 55.8%, 74.4%, 83.6%, and 80.7% of the Mackinder’s owl diet in the four valleys of Mt. Kenya, respectively. *Otomys orestes/tropicalis* have been trapped frequently, with many of their tracks found among the tussocks in the Sirimon area (Coe & Foster, 1972). In contrast, Ogada (2008) found that Mackinder’s owl near farmlands took a variety of prey species, unlike results from other studies (Coe, 1967; Coe & Foster, 1972; Rödel et al., 2002; Sessions, 1972) and suggested that her finding supports the optimum foraging theory that more productive environments have predators with more specialized diets, whereas patchy environments contain generalist predators. Mackinder’s owl takes a variety of prey but the most abundant at its roost site dominates its diet (Sessions, 1972). *Otomys* sp. are found in large numbers in the alpine zone in the tussock grassland and lake edges, and Coe (1967) reported that Mackinder’s owl feeds almost exclusively on them. This suggests that *Otomys* sp. are more common in Sirimon than in Chogoria as predators tend to consume more of the available prey (Davis et al., 2015; Geffen et al., 1992; Tilson et al., 1980). Coe (1967) reported that the rock hyrax (*Procavia* sp.) common duiker (*Sylvicapra grimmia altivallis*) and groove toothed rat (*O. orestes*) are the main herbivores of the Sirimon alpine zone of Mt. Kenya, but that *Rhabdomys* and *Lophuromys* are also present, with *T. splendens* found up to 3 800 m a.s.l. and all were found in the scats/pellets we collected. *Graphiurus, Crocidura alex alpina*, and *Crocidura f. fumosa* have been recorded/caught in the Sirimon area (Coe & Foster, 1972) but *Graphiurus* sp. remains were not identified in any of the scats we collected. The *Otomys* sp. found during the study was not determined to species because the distribution of *O. orestes* and *O. tropicalis* overlap in Mt. Kenya, as per Coe & Foster (1972), or are considered synonymous in Happold (2013).

In conclusion, analysis of prey remains in carnivore scats can give important information on the diet of carnivores and the abundance of prey in a given area. However, some prey (especially those whose remnants are completely digested) may remain undetected using comparative morphological identification techniques. For example, Sillero-Zubiri et al. (1995) observed a higher frequency of larger prey consumption in Ethiopian wolves than was determined in the scat remains analysis. In contrast, in the same study, Sillero-Zubiri et al. (1995) found that the giant mole rat frequency of occurrence in scats was higher than that by direct observations. This underscores the importance of scat analysis in revealing cryptic prey of a carnivore in an area but also that comprehensive study should combine scat, hair, and bone remains analysis with standard surveys. Furthermore, while some predators’ feces can be identified due to their unique characteristics, some are very similar (especially those of smaller carnivores) and can be difficult to distinguish. We therefore recommend the use of camera traps and possibly DNA in the future to aid in documenting predator diversity in the study areas.
